# Complex crosstalk: early-onset lupus and antiphospholipid syndrome in a child with *SHOC2*-related RASopathy

**DOI:** 10.1093/rheumatology/keaf144

**Published:** 2025-03-14

**Authors:** Bengisu Menentoğlu, Esma Nur Konur Akbaş, Gülşah Kavrul Kayaalp, Özlem Akgün, Ayça Dilruba Aslanger, Zehra Oya Uyguner, Nuray Aktay Ayaz

**Affiliations:** Division of Pediatric Rheumatology, Department of Pediatrics, Istanbul Faculty of Medicine, Istanbul University, Istanbul, Türkiye; Department of Medical Genetics, Istanbul Faculty of Medicine, Istanbul University, Istanbul, Türkiye; Division of Pediatric Rheumatology, Department of Pediatrics, Istanbul Faculty of Medicine, Istanbul University, Istanbul, Türkiye; Division of Pediatric Rheumatology, Department of Pediatrics, Istanbul Faculty of Medicine, Istanbul University, Istanbul, Türkiye; Department of Medical Genetics, Istanbul Faculty of Medicine, Istanbul University, Istanbul, Türkiye; Department of Medical Genetics, Istanbul Faculty of Medicine, Istanbul University, Istanbul, Türkiye; Division of Pediatric Rheumatology, Department of Pediatrics, Istanbul Faculty of Medicine, Istanbul University, Istanbul, Türkiye

Rheumatology key message

- RASopathies are a rare monogenic cause of early-onset lupus, with their association with antiphospholipid syndrome being previously unrecognized.

Dear Editor, Childhood-onset systemic lupus erythematosus (cSLE) is a chronic, potentially life-threatening, multisystem autoimmune disorder diagnosed in individuals below the age of 18 years [1]. It is generally marked by a more rapid progression and regrettably exhibits a greater frequency of significant organ involvement compared with adult-onset varieties. The aetiology of the disease is complex and not fully elucidated, its pathogenesis is characterized by the production of antibodies against nuclear and cytoplasmic antigens, immune complex deposition and activation of complement pathways. Contemporary research indicates that a genetic predisposition may play a crucial role in pathogenesis. The review by E.Y. Chan *et al.* emphasized the importance of suspecting and thoroughly evaluating monogenic forms of SLE in at-risk patients, as targeted treatments may be available [2]. Recent investigations have revealed that specific pathogenic variations associated with RASopathies are also linked to monogenic variants of lupus [3]. A notable example is Noonan-like syndrome with RAS signalling dysregulation, which is caused by a recurrent missense alteration in the *SHOC2* gene. Activation of the RAS/MAPK signalling pathway within immune cells further exacerbates the development of autoimmune conditions such as SLE [4]. This report presents a rare case of monogenic lupus with Noonan-like syndrome characterized by a distinctive initial clinical presentation.

A two-year-old girl presented to the pediatric emergency unit with reduced muscular strength in both left upper and lower extremities, accompanied by significant difficulties in ambulation. She had no prior complaints and had been followed by cardiology since one month of age for pulmonary stenosis and an atrial septal defect.

Physical examination revealed left hemiparesis. Cranial magnetic resonance imaging demonstrated a watershed infarct that aligns with the vascular territories supplied by the anterior cerebral artery and the middle cerebral artery (Fig. 1). Complement levels were noted to be decreased [C3 64 mg/dl (85–160 mg/dl) and C4 2.3 mg/dl (12–36 mg/dl)]. Positive ANA with titre 1/640 and homogeneous pattern (normal: <1/80), anti-double-stranded DNA levels at 124 IU/ml (normal: <100 IU/ml). Additionally, antiphospholipid IgG was elevated at 80 GPLU/ml (normal: <12 GPLU/ml) and both lupus anticoagulant and the direct Coombs test were positive. Albumin level was 4.2 g/dl (normal: 3.0–5.3 g/dl) and complete blood count revealed no cytopenia. Other biochemical parameters were normal. Pleural effusion and ascites were detected on ultrasonography. A 24-h urine analysis revealed 20 mg/m^2^/h proteinuria (normal: <4 mg/m^2^/h). The renal biopsy was not performed due to the lack of consent. During the follow-up, proteinuria regressed spontaneously. The patient was short in stature, had dysmorphic features, loose anagen hair and a webbed neck, as shown in detail in Fig. 1.

**Figure 1.**
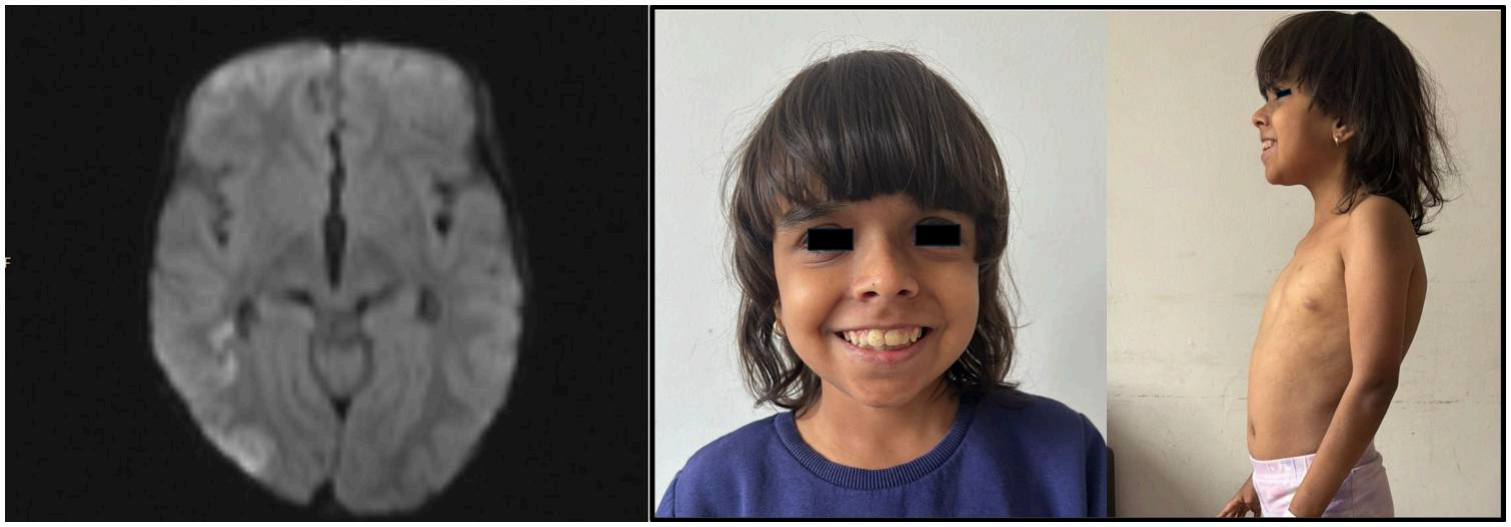
MRI image and dysmorphic features of the patient. Diffusion restriction in the right hemisphere consistent with watershed infarct on MRI and the appearance of the patient at the age of 13 years is remarkable for dysmorphic findings; darkly pigmented skin, coarse hair, rugged facial morphology, hypertelorism, inferiorly positioned palpebral fissures, prominent infraorbital lines, depressed nasal root, low-set ears, micrognathia, pointed chin and pectus excavatum

The patient fulfilled both the 1997 American College of Rheumatology criteria and 2012 Systemic Lupus International Collaborating Clinics criteria, leading to a diagnosis of SLE. The patient received methylprednisolone at a dose of 30 mg/kg/day for five days, followed by a dose of 2 mg/kg/day. Cyclophosphamide (750 mg/m^2^/day) was administered monthly for six doses, after which maintenance with mycophenolate mofetil (600 mg/m^2^/day) was initiated. Additionally, hydroxychloroquine and acetylsalicylic acid were prescribed for antiphospholipid syndrome, while enalapril was commenced for hypertension and proteinuria. Genetic analysis was performed due to early onset disease and current dysmorphic findings, which revealed the *SHOC2* variant (NM_007373.4: c.4A>G/p.(Ser2Gly)/rs267607048). During follow-up evaluations, the patient exhibited recurrent manifestations of arthritis, as well as pleural and pericardial effusions.

Recent studies have identified several genetic variants associated with SLE. Presently, mutations across >50 distinct genes associated with complement pathways (such as C1, C2, C4), type I interferon signalling (including DNASE, RNASE, TREX, IFIH1, ADAR), mechanisms of self-tolerance (for example, PRKCD, TNFSF13B) or RAS signalling pathways (like PTPN11, SOS1, KRAS, SHOC2) are indicated in the aetiology of monogenic lupus [2].

RASopathies are a group of neurodevelopmental disorders that include Noonan syndrome and related syndromic conditions. Individuals with these disorders often exhibit the classic phenotype along with autoimmune manifestations such as SLE, autoimmune thyroiditis, vitiligo and celiac disease [5]. Key features commonly associated with RASopathies include short stature, craniofacial anomalies, webbed neck, cardiac abnormalities, variable cognitive impairments and an increased risk of cancer development [6].

A total of 13 patients with RASopathy and SLE have been reported in the literature, five variants associated with *SHOC2*, two with *KRAS*, one with *PTPN11* and five remained genetically undiagnosed [3, 7–9]. Unlike classical SLE, the lower frequency of skin involvement and the higher rate of serositis are notable, which closely resembles presentation in our patient. Lupus nephritis has been documented in only one previously reported case of monogenic lupus associated with an *SHOC2* alteration, and renal involvement was not a prominent feature in our patient [9].

RASopathies are a rare cause of monogenic lupus, and their association with antiphospholipid syndrome, as seen in our patient, is unprecedented. Monogenic lupus should be considered in cases with dysmorphic features, male gender, family history or early-onset disease, and genetic analysis is recommended in such situations.

Informed consent was obtained for the publication of this article.

## Data Availability

All data associated with this paper are available, and data not presented in the manuscript can be accessed upon request.
